# Ionic Liquid-Dispersive Micro-Extraction and Detection by High Performance Liquid Chromatography–Mass Spectrometry for Antifouling Biocides in Water

**DOI:** 10.3390/molecules28031263

**Published:** 2023-01-28

**Authors:** Li Zhou, Tong Wu, Chuanshan Yu, Shaowen Liu, Canping Pan

**Affiliations:** 1 College of Science, China Agricultural University, Beijing 100193, China; 2 Tea Research Institute, Chinese Academy of Agricultural Sciences, Hangzhou 310008, China

**Keywords:** antifouling biocides, high-performance liquid chromatography–mass spectrometry (LC-MS), ionic liquid-dispersive liquid–liquid micro-extraction (IL-DLLME)

## Abstract

A simple analytical method was developed and evaluated for the determination of two antifouling biocides using an ionic liquid-dispersive liquid–liquid micro-extraction (IL-DLLME) and a high-performance liquid chromatography–electrospray ionization mass spectrometry (LC-ESI-MS) analysis. Irgarol 1051 and Sea-Nine 211 were extracted from deionized water, lake water, and seawater using IL 1-hexyl-3-methylimidazolium hexafluorophosphate ([HMIm][PF6]) and ethyl acetate as the extraction solvent and the dispersion solvent. Several factors were considered, including the type and volume of extraction and dispersive solvent, IL amount, sample pH, salt effect, and cooling temperature. The developed method resulted in a recovery range of 78.7–90.3%, with a relative standard deviation (RSD, *n* = 3) less than 7.5%. The analytes were enriched greater than 40-fold, and the limits of detection (LOD) for two antifouling biocides were 0.01–0.1 μg L^−1^. The method was effectively applied for the analysis of real samples of freshwater as well as samples of seawater.

## 1. Introduction

The marine industry is hindered by marine biofouling, which damages submerged equipment and raises production costs. Antifouling biocides are commonly employed to prevent the attachment of fouling organisms to ships and other equipment [1]. Since harmful antifouling paints containing tributyltin (TBT) have been banned [2], new organic booster biocides have become the main constituents of antifouling paints to enhance their efficacy. These biocides include metal-based compounds such as zinc pyrithione and zineb, as well as non-metallic compounds such as Irgarol 1051, Sea-nine 211, Kathon 5287, chlorothalonil, dichlofluanid, and thiram [3]. However, the use of these compounds appears to be hazardous due to their residues, toxicity, and resultant contamination of the aquatic environment, as well as the potential impact on public health. Irgarol 1051 is highly toxic to non-target marine algae [4], as it destabilizes aquatic herbivorous mammal populations [5] and causes coral bleaching [6]. The use of Irgarol 1051 in antifouling paints is restricted in the European Union and the United States [7,8]. Despite having a significantly better environmental profile, Sea-Nine 211 is still hazardous to fish [9], sea urchins, and embryos [10].

Recently, these antifouling biocides have been widely identified in marinas and harbors throughout the world [11]. In aquatic environments, concentrations of Irgarol 1051 ranged from 0.12–4800 ng L^−1^ [12,13], whereas concentrations of Sea-Nine 211 ranged from 0.1–3300 ng L^−1^ [14,15]. Due to their prevalence at low concentrations, pre-concentration techniques and sensitivity detection are generally highlighted. Most analysis strategies in recent years have been based on liquid–liquid extraction (LLE) [16], solid-phase extraction (SPE) [17,18], and a few others, including solid-phase microextraction (SPME) [19], stir bar sorptive extraction and thermal desorption (SBSE-TD) [20], and microfunnel-supported liquid-phase microextraction (MF-LPME) [21]. In terms of the examination of antifouling biocides, the aforementioned approaches have various drawbacks, such as being tedious, time-consuming, expensive, complex, and harmful to the environment. Therefore, the development of less complex, more effective, and safe extraction approaches for the identification of antifouling biocides is receiving a lot of focus.

Dispersive liquid–liquid micro-extraction (DLLME) is a simple, rapid, inexpensive, sensitive, and effective technique for the extraction of target analytes. According to the DLLME principle, a water sample containing target analytes is quickly mixed with a mixture of an extraction solvent and a dispersion solvent to produce a ternary component solvent system, in which the target analytes are enriched into the micro-extraction solvent [22]. Organic solvents with a high density, incompatibility in water, and high solubility for target analytes are commonly utilized as extraction solvents. Organic solvents that are miscible with the extraction solvent and water are employed as disperser solvents to assist the extraction solvent in forming dispersed micro-droplets in the water sample, hence increasing the contact area between the extraction solvent and the target analytes [23]. Traditional DLLME employs highly toxic extraction solvents such as chlorobenzene, carbon tetrachloride, chloroform, dichloro-methane, and tetrachloroethylene [24,25]. The DLLME technique is currently being improved by employing low toxicity and new extraction solvents [25]. Since the majority of target analytes are polar compounds, the ideal DLLME extraction solvents must be liquid under standard conditions, have a low vapor pressure, be incompatible with water, have a high polarity, and have a high density.

Ionic liquids (ILs) are organic salts with melting points lower than 100 °C, composed of organic cations and organic or inorganic anions [22]. ILs have distinctive characteristics, including high thermal stability, low vapor pressure, high viscosity, and low toxicity [26]. Particularly, their physicochemical properties can be modified by selecting a particular combination of anions and cations to enhance the solubility of specific analytes [27]. Therefore, various ILs have been used as extraction solvents of DLLME, such as 1-hexyl-3-methylimidazolium hexafluorophosphate ([HMIM][PF6]), 1-butyl-3-methylimidazolium hexafluorophosphate, tetradecyl (trihexyl) phosphonium chloride, and 1-butyl-3-methylimidazolium bis (trifluoromethylsulfonyl) imide [23,28], which are typically recognized as green solvents in analytical chemistry [29], thereby deriving the IL-DLLME approach. Neurotransmitters [30], anthraquinones [31], phthalate esters [32], organic dyes [33], metal ions [34,35], pesticides [36,37], antibiotics [38,39], and other biological compounds, as well as food and environmental pollutants, have all been focused using IL-DLLME. However, the IL has not yet been used to extract antifouling biocides from water samples.

In this study, the potential application of IL-DLLME and LC–MS for the identification of two kinds of antifouling biocides in water samples was investigated. An IL ([HMIm][PF6]) was used as the extraction solvent, whereas ethyl acetate was selected as the dispersion solvent. The effects of various experimental factors on the extraction were examined, and the process was validated via linearity, precision, and accuracy investigations. The developed method can be used to analyze real lake water and seawater samples.

## 2. Results and Discussion

### 2.1. Optimization of IL-DLLME Procedure

All parameters of IL-DLLME conditions were optimized using deionized water (5.0 mL) spiked with antifouling biocides. Each data point was determined using the mean of three separate extractions.

#### 2.1.1. Effect of Amount of IL

**Figure 1:**
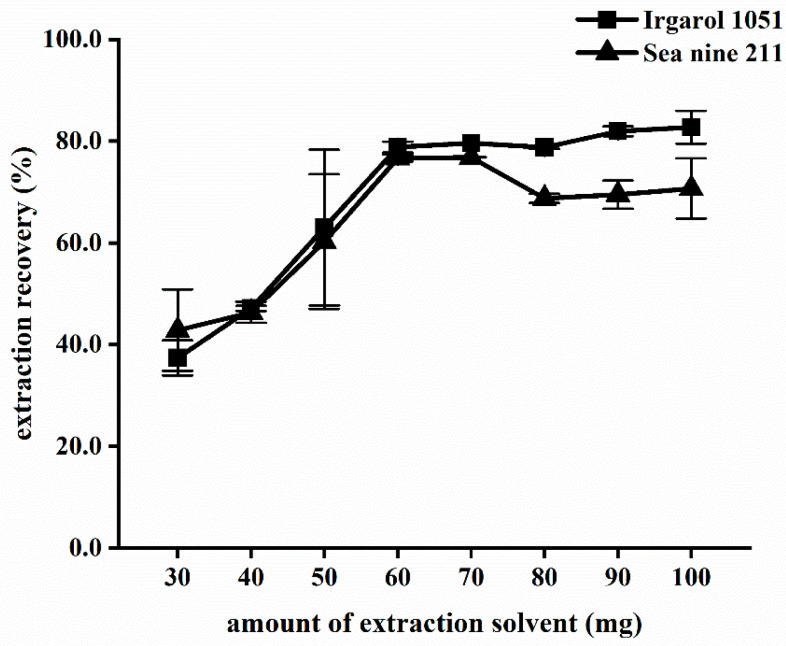
Effect of amount of [HMIm][PF6] on extraction recovery. Extraction conditions: water sample, 5.0 mL; disperser solvent, ethyl acetate 0.4 mL; NaCl 0% (*w*/*v*).; pH 6; and cooling temperature 20 °C.

Because of its low water solubility, low volatility, and higher density than water, [HMIm][PF6] has been widely used as an extraction solvent for pesticides [40], metal ions [41], mycotoxins [24], and polycyclic aromatic hydrocarbons [42]. Quantities of 30 mg, 40 mg, 50 mg, 60 mg, 70 mg, 80 mg, 90 mg, and 100 mg of [HMIm][PF6] were analyzed in deionized water that was spiked with 2 μg L^−1^ of Irgarol 1051 and 10 μg L^−1^ of Sea-Nine 211 at a constant volume of disperser solvent (0.4 mL) (Figure 1). As the amount of IL increased from 30 to 60 mg, the recoveries exhibited a similar linear sign increase. However, when the amount of IL exceeded 60 mg, the recoveries declined or remained nearly constant. Wang et al. discovered that when [HMIm][PF6] exceeded 60 μL in their study on the analysis of fungicides in fruit juice, the recoveries decreased [43]. The distribution coefficient and recovery of analytes in IL may have been reduced as a result of the larger amounts of IL being dissolved, which could have decreased the polarity of the aqueous phase [44]. The optimal amount of IL was therefore determined to be 60 mg.

#### 2.1.2. Selection of Disperser Solvent and Effect of Volume

The disperser solvent must be miscible with the extraction solvent and the water sample, thereby increasing the contact area and interaction between the two phases to enhance the extraction efficiency. The selection of a disperser is crucial for achieving excellent preconcentration and extraction effects. Consequently, four potential disperser solvents, acetone, methanol, acetonitrile, and ethyl acetate, were tested. The sample solutions for this, and the subsequent tests used 5 mL of deionized water spiked with 1 μg L^−1^ of Irgarol 1051 and 5 μg L^−1^ of Sea-Nine 211. A series of sample solutions were analyzed using 0.5 mL of each disperser solvent containing 60 mg of [HMIm][PF6]. The results showed that Irgarol 1051 (85.0%) and Sea-Nine 211 (86.0%) had higher recoveries when ethyl acetate was used as the dispersant than those of acetone (Irgarol 1051 33.6%, Sea-Nine 211 50.1%), methanol (Irgarol 1051 36.4%, Sea-Nine 211 45.8%), and acetonitrile (Irgarol 1051 61.9%, Sea-Nine 211 62.8%). Kong et al. also examined vitamins and carotenoids in human serum using ethyl acetate as the disperser solvent [45]. The use of ethyl acetate as the disperser solvent resulted in good media miscibility and the best recoveries. As a result, ethyl acetate was selected for further investigation.

**Figure 2:**
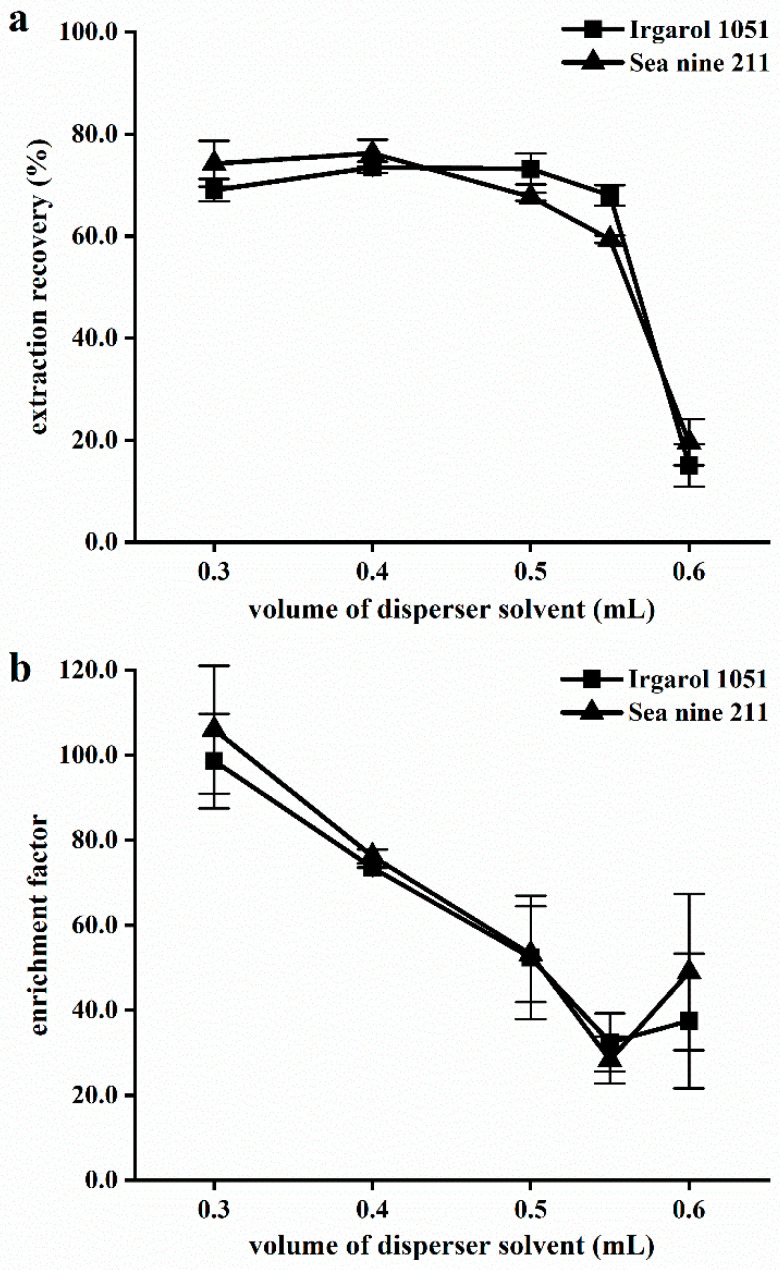
Effect of ethyl acetate volume on (**a**) extraction recovery and (**b**) enrichment factor. Extraction conditions: water sample, 5.0 mL; extraction solvent [HMIm][PF6] 60 mg; NaCl 0% (*w*/*v*).; pH 6; and cooling temperature 20 °C.

The volume of the disperser affects the dispersion degree of the extraction phase in the aqueous phase, thereby influencing the extraction efficiency. When the disperser volume is small, the extraction solvent cannot be completely dispersed in the aqueous phase, preventing the formation of a good ternary cloudy solution of water/disperser/extraction solvent, and lowering the extraction efficiency. In contrast, when the volume of the disperser is increased, the distribution coefficient of analytes in the water rises, and the extraction efficiency decreases. To assess the impact of the organic solvent on the yield of the IL-DLLME process, various ethyl acetate volumes were tested. To determine the optimal volume, experiments were conducted with varying volumes of ethyl acetate (0.30 mL, 0.40 mL, 0.50 mL, 0.55 mL, and 0.60 mL) mixed with 60 mg [HMIm][PF6]. Figure 2 shows that, in contrast to the enrichment factor (EF), the recoveries increased initially and then decreased as the volume of ethyl acetate increased. A total of 0.4 mL of ethyl acetate yielded the highest recoveries for all analytes. Similar behavior was observed when parabens were analyzed using IL-DLLME [46]. This can be explained by the possibility that if there is insufficient dispersion solvent, the extraction solvent may not make good contact with the analytes in the sample solution, which could lower the recovery. On the other hand, more disperser solution resulted in a more settled phase, which decreased the EF. The results showed that 0.4 mL was selected to achieve a high EF and a good extraction recovery (ER).

#### 2.1.3. Salt Effect 

**Figure 3:**
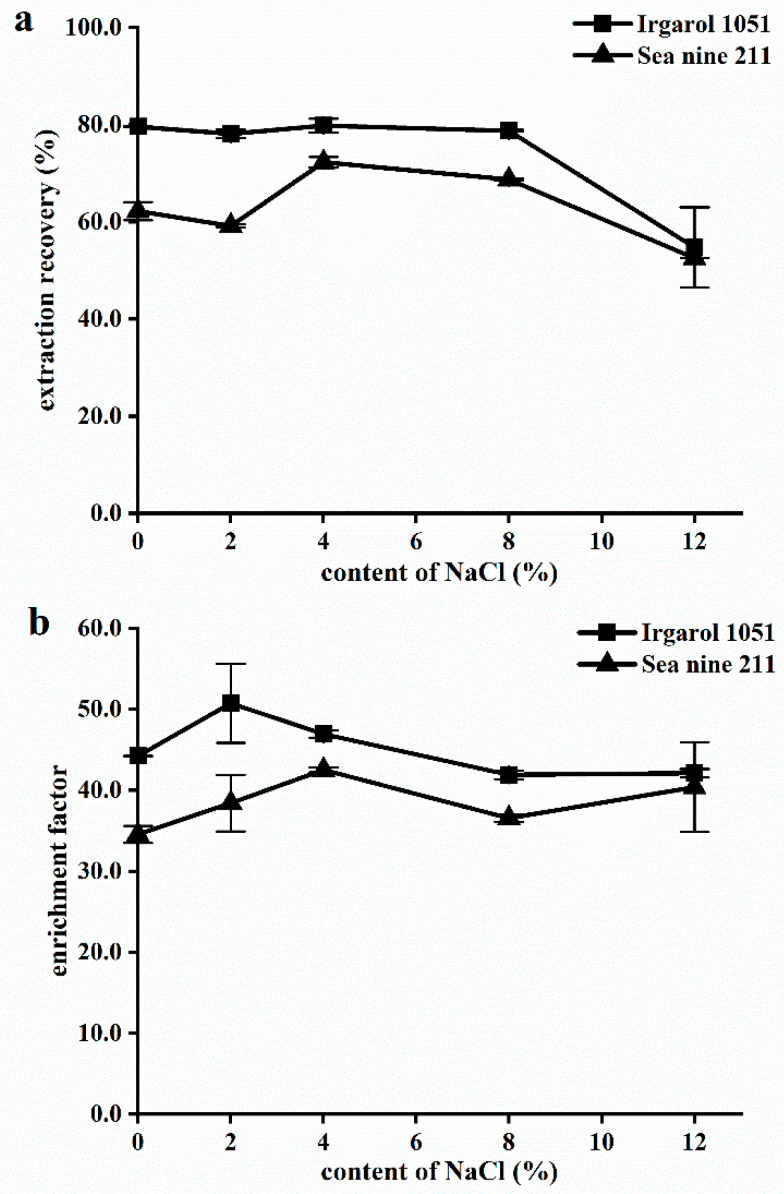
Influence of content of NaCl on (**a**) extraction recovery and (**b**) enrichment factor. Extraction conditions: water sample, 5.0 mL; disperser solvent, ethyl acetate 0.4 mL; extraction solvent [HMIm][PF6] 60 mg; pH 6; and cooling temperature 20 °C.

In general, an increase in ionic strength frequently results in better extraction performance with salting out, which has an impact on the analyte partitioning coefficients between the aqueous and organic phases. In contrast, the addition of salt increases the ionic liquid’s solubility in water, resulting in low recovery [47]. Different NaCl concentrations (0%, 2%, 4%, 8%, and 12%, *w*/*v*) were added to deionized water to assess the impact of the ionic strength on the effectiveness of extraction and enrichment. As depicted in Figure 3, the addition of salt had no discernible effect on either the EF or ER at concentrations of NaCl less than 8%. With a higher concentration and an increase in ILs solubility in the aqueous phase, the sediment volume decreased, resulting in a low ER and a high EF. In the study that used 1-octyl-3-methylimidazolium hexafluorophosphate ([C8MIM][PF6]) to extract pyrethroid pesticides, Zhang et al. also discovered that a high salt concentration increased the viscosity of the water phase and improved the solubility of IL in water, thereby reducing the extraction efficiency [48]. As a result, no NaCl was added to the water samples, allowing the proposed method to be used for the preconcentration of Irgarol 1051 and Sea-Nine 211 in both fresh and salty water.

#### 2.1.4. Sample pH

**Figure 4:**
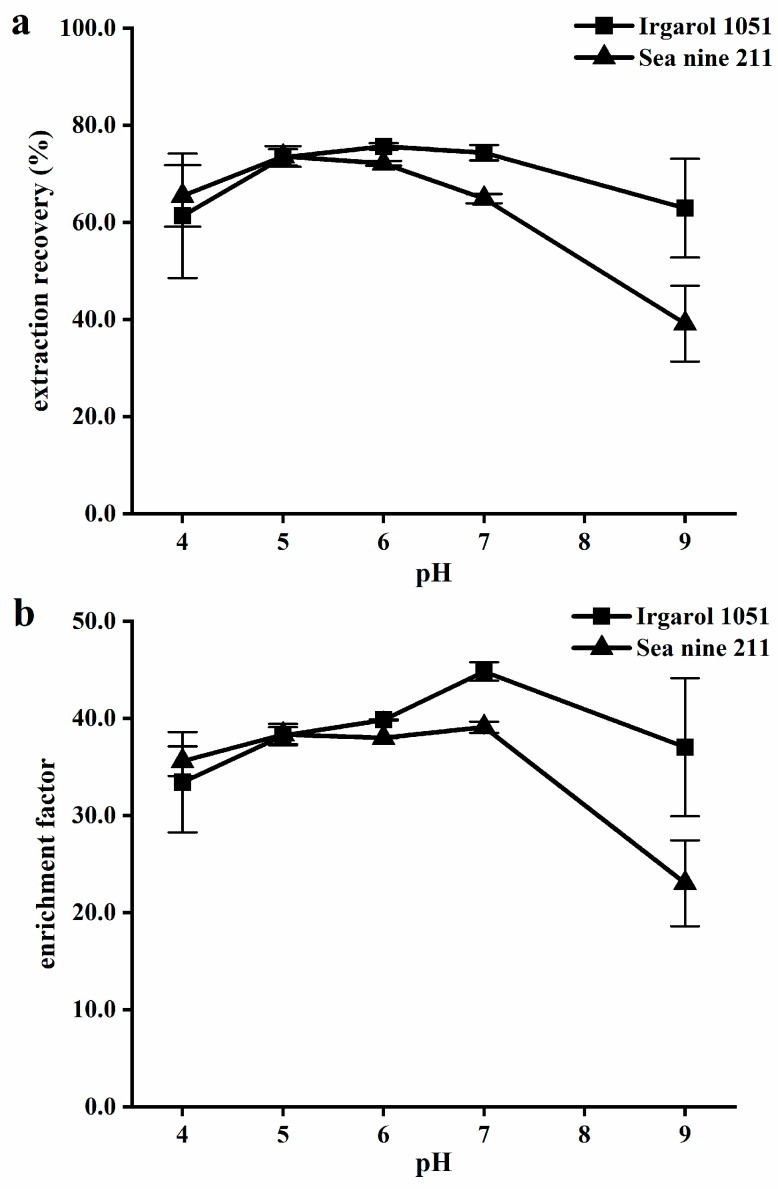
Influence of pH on (**a**) extraction recovery and (**b**) enrichment factor. Extraction conditions: water sample, 5.0 mL; disperser solvent, ethyl acetate 0.4 mL; extraction solvent [HMIm][PF6] 60 mg; NaCl 0% (*w*/*v*); and cooling temperature 20 °C.

The effect of various pH levels (4, 5, 6, 7, and 9) on IL-DLLME ER and EF was examined by adding the appropriate amount of hydrochloric acid or sodium hydroxide solution to water samples. The results are displayed in Figure 4, which shows that pH 5 or pH 6 provided the best analyte recovery. Similar behavior was observed in a prior study that used IL-DLLME to identify organophosphorus pesticides [40]. The results indicated that Irgarol 1051 (p*K*_a_ 4.13 ± 0.10) and Sea-Nine 211 (p*K*_a_ −6.09 ± 0.60) were relatively stable and had a high IL distribution coefficient in neutral and weakly acidic media, and that they could be decomposed in strong bases. A pH of 6 was selected due to the ease of operation. Since the pH of the utilized deionized water was approximately 6, pH adjustments were avoided throughout the entire optimization procedure. After being diluted with deionized water, the real water samples were examined. 

#### 2.1.5. Effect of Cooling Temperature

**Figure 5:**
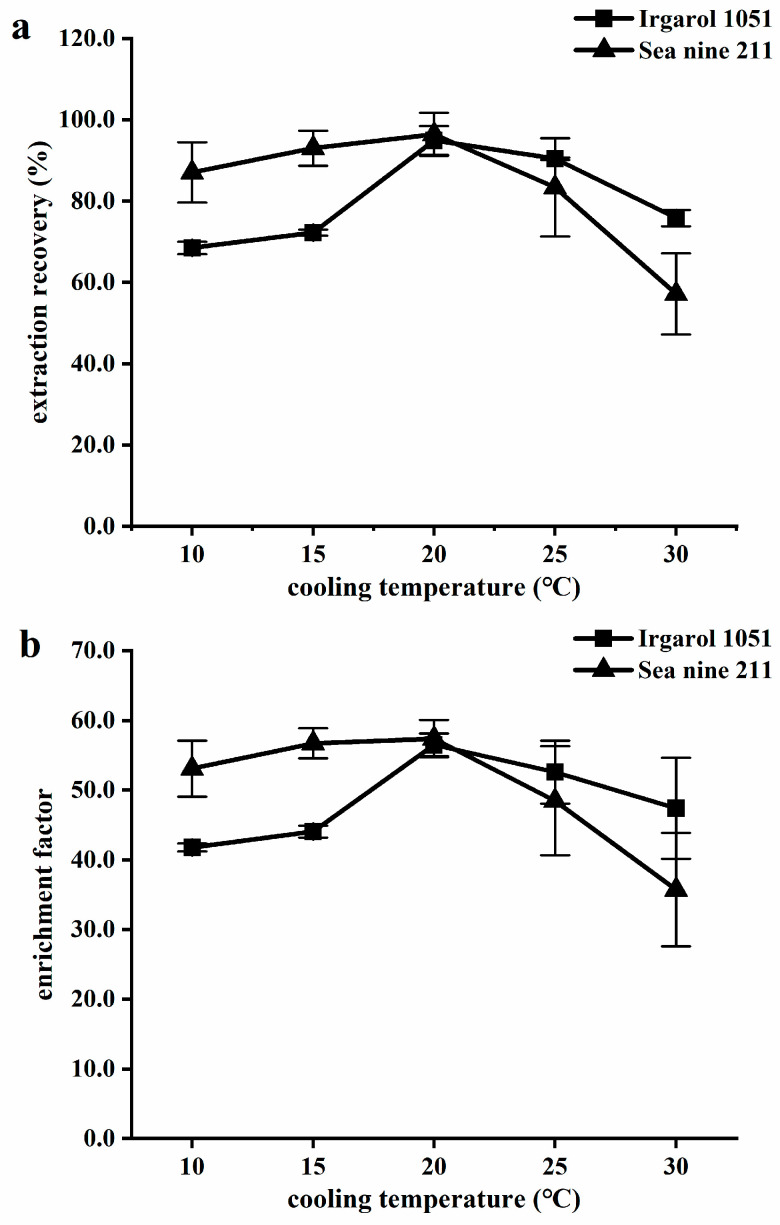
Influence of cooling temperature on (**a**) extraction recovery and (**b**) enrichment factor. Extraction conditions: water sample, 5.0 mL; disperser solvent, ethyl acetate 0.4 mL; extraction solvent [HMIm][PF6] 60 mg; NaCl 0% (*w*/*v*); and pH 6.

Temperature can influence analyte partition coefficients, IL solubility in water, and phase separation [49]. The different cooling temperatures (10 °C, 15 °C, 20 °C, 25 °C, and 30 °C) in the water bath (defined as the temperature before centrifugation and after extraction) were investigated at 30 °C of the extraction temperature. As shown in Figure 5, as the temperature decreased from 30 °C, the recovery initially increased and then reduced. In varying temperatures, the EF exhibited the same characteristics as the ER. Therefore, it can be concluded that the partition coefficient of analytes between IL and water had a significant impact on recovery and enrichment. The cooling temperature was found to have the greatest contribution of all the optimized factors. The recovery of Irgarol 1051 increased from 75.8% to 94.9%, while the recovery of Sea-Nine 211 increased from 57.2% to 96.4%, with a decrease in temperature from 30 °C to 20 °C. In the following method validation studies, 20 °C was used.

### 2.2. Method Validation

**Figure 6:**
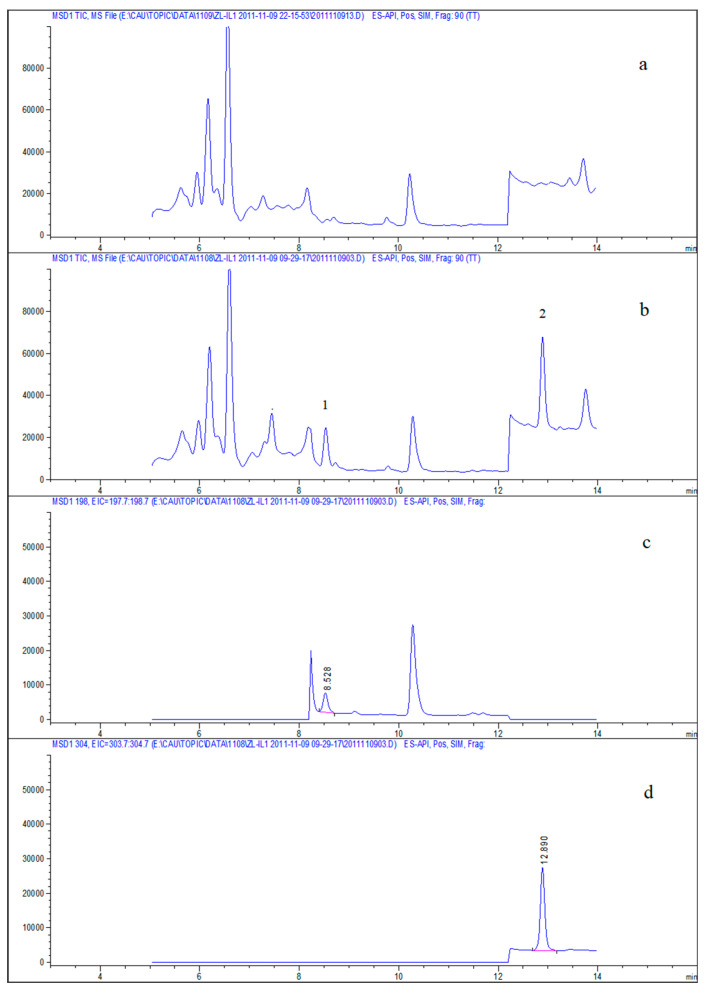
Chromatograms of analytes spiked in the water sample. (**a**) Sum chromatogram of blank, (**b**) sum chromatogram of spiked water, (**c**) extracted ion chromatogram (EIC) of ion 198, and (**d**) EIC chromatogram of ion 304. 1-Irgarol 1051, spiked 0.02 μg L^−1^; 2-Sea-Nine 211, spiked 0.12 μg L^−1^.

To validate the analytical approach, the series levels of spiked samples in deionized water, lake water, and seawater were examined (Table 1). Linearities were determined using deionized water spiked with five different concentrations of Irgarol 1051 (0.02 μg L^−1^, 0.2 μg L^−1^, 2 μg L^−1^, 20 μg L^−1^, and 100 μg L^−1^) and Sea-Nine 211 (0.1 μg L^−1^, 1 μg L^−1^, 10 μg L^−1^, 100 μg L^−1^, and 500 μg L^−1^). Calibration curves exhibited the linear relationships between analyte peak regions and concentrations. The equations for the calibration curves of Irgarol 1051 and Sea-Nine 211 were y = 70,515,778x − 32,368 and y = 11,067,977x + 53,400, respectively, and their respective correlation coefficients (R^2^) were 0.9995 and 0.9993. The accuracy and precision of this method were validated using a recovery experiment. Analytes were spiked at three concentration levels in deionized water, lake water, and seawater samples, respectively, and each concentration level was repeated in triplicate. The mean recoveries ranged from 78.7% to 90.3%, and all relative standard deviations (RSDs) were less than 7.5%. The accuracy and precision of this method met the requirements for reliable analyte detection (recoveries were 70–120%, RSD < 20%) [50]. The limits of detection (LOD) and quantification (LOQ) were determined as the analyte concentrations corresponding to the instrument responses of 3 and 10 signal/noise, respectively, by injecting spiked samples of deionized water, lake water, and seawater. This method had LODs and LOQs of 0.01–0.1 μg L^−1^ and 0.02–0.5 μg L^−1^, respectively, with the EF ranging from 22 to 45. Figure 6 depicts a typical chromatogram of antifouling biocides in a spiked water sample.

### 2.3. Real Water Samples Analysis

Finally, the developed analytical methodology was evaluated for its practical application in extracting antifouling biocides from freshwater and seawater. The environmental risk limit (ERL) is the concentration level at which pollutants pose a possible threat to the environment. The previous literature revealed the 0.024 μg L^−1^ ERL for Irgarol 1051 in water [51]. According to the European Union directive, the maximum allowable concentration of environmental quality standards (EQS) for Irgarol was 0.016 μg L^−1^ in water [52]. The limit standard for Sea-Nine 211 is still undefined. The suggested IL-DLLME technique has LODs 0.01–0.1μg L^−1^. Therefore, this method typically achieved the criteria for detecting antifouling biocides from real water samples. The freshwater was collected from the North Sea Lake and Xiaoqing River in the city of Beijing, China, while the seawater was collected from Qing Dao, China. The outcomes revealed that the examined water samples were well below the LODs of the proposed method. Therefore, the antifouling biocides did not represent a significant threat to the aquatic ecosystem described above.

### 2.4. Comparison of IL-DLLME with Other Sample Preparation Techniques

Table 2 represents the performance of the proposed IL-DLLME approach in comparison to existing reported extraction procedures for the determination of antifouling biocides in water samples, such as LLE, SPE, SPME, SBSE, and LPME. Large sample volumes and substantial enrichment are responsible for the drastically reduced LOD obtained using SPE and LLE techniques. However, the enormous number of samples results in a prolonged extraction time and considerable consumption of organic solvent. IL-DLLME only requires a small amount of sample and organic solvent for extraction, and its recovery and RSD values are comparable to those of SPE and LLE. The extraction solvent is not necessary for LPME, SBSE, or SPME; however, these processes require a long time and requirements for specialized equipment. With a lower LOD than the SPME approach, the simple operation of the IL-DLLME procedure facilitates the whole sample treatment; just a few minutes are required before instrument analysis. All of these results indicate that the optimized IL-DLLME procedure appears to be a reproducible, rapid, simple, and low-cost alternative that can be used for the preconcentration of antifouling biocides such as Irgarol 1051 and Sea-Nine 211 from water samples.

## 3. Materials and Methods

### 3.1. Reagents and Chemicals

Analytical standards for Irgarol 1051 were supplied by Dr. Ehrenstorfer (Augsburg, Germany), and Sea-Nine 211 was supplied by Pure Chemistry Scientific Inc. (Newton, MA, USA). The basic information about analytes is detailed in Table 3. The standard stock solution of 1 mg mL^−1^ was prepared in acetonitrile. The stock solution was diluted with acetonitrile to provide a working standard solution of 10 μg mL^−1^. Both standard stock solutions and working solutions were stored at −20 °C. HPLC-grade acetonitrile, methanol, and ethyl acetate (Fisher Scientific, Waltham, MA, USA) were used. IL [HMIm][PF6] was acquired from the Lanzhou Institute of Chemical Physics of Chinese Academy of Sciences (Lanzhou, China). Sodium chloride (NaCl, AR) was purchased from Sinopharm Beijing Chemical and Reagent Ltd. (Beijing, China). Deionized water (18 M/cm) was prepared by a MILI-Q Pure treatment system (Millipore, St. Louis, MO, USA). The freshwater was collected from the North Sea Lake, an artificial lake in the city of Beijing. The seawater was collected from the Yellow Sea.

### 3.2. Apparatus

The analytes were separated from the extracts using the Agilent 1260 series HPLC (Agilent Technologies, Palo Alto, CA, USA). A ZORBAX SB-C18 column (150 mm × 4.6 mm i.d., 3.5 μm; Agilent Technologies, Palo Alto, CA, USA) was employed. The mobile phase was comprised of methanol (A) and 0.1% formic acid in water (B). The gradient program was as follows: 0–5 min, 55–85% A; 5–7 min, 85% A; 7–10 min, 85–95% A; 10–13 min, 95% A; 13–15 min, 95–55% A; and 15–19 min, 55% A. The flow rate was 0.6 mL min^−1^ and the injection volume was 10 μL. The column temperature was maintained at 30 °C.

The HPLC system was coupled to an Agilent 6130 Single Quadrupole mass spectrometer equipped with an electrospray source in positive ionization mode. The operational parameters were as follows: drying gas flow 10.0 L min^−1^, drying gas temperature 350 °C, nebulizer gas pressure 35 psi., and capillary voltage 3000 V. Flow injection analysis (FIA) was used to optimize the fragmentor, and analytes were quantified in the selected ion monitoring mode (SIM). The chromatographic parameters of the analytes are presented in Table 3.

### 3.3. IL-DLLME Procedure

The environmental samples, including lake water and seawater, were filtered with 0.45 μm water phase membrane prior to analysis. After that, the seawater had to be diluted fourfold with deionized water. In a 15 mL conical-bottomed centrifuge tube, 5.0 mL of water samples were placed. The aqueous phase was then rapidly injected with 60 mg of the [HMIm][PF6] and 0.4 mL of ethyl acetate as extraction and disperser solvents, followed by 1 min of manual shaking. After cooling the cloudy solution in a 20 °C water bath and centrifuging at 3800× *g* rpm for 5 min, the IL phase settled at the bottom of the tube. The IL phase was collected and diluted with acetonitrile to a final volume of 150 μL after the upper aqueous phase had been removed using a syringe.

## 4. Conclusions

This research used an IL-DLLME methodology coupled with LC-MS to identify two types of commonly used booster biocides in water samples. The quantity of the IL ([HMIm][PF6]) utilized as an extraction solvent for Irgarol 1051 and Sea-Nine 211 was first optimized. Furthermore, the type and volume of the disperser solvent, the amount of salt, the pH, and the cooling temperature were studied to determine the optimal extraction conditions. A systematic validation demonstrated that the proposed method has acceptable linearity (R^2^ > 0.999), recovery (78.7–90.3%), and repeatability (RSD ≤ 7.5%). The LOD and LOQ of this method were found to be 0.01–0.1 μg L^−1^ and 0.02–0.5 μg L^−1^, respectively. The successful utilization of lake water and seawater samples revealed that the method is acceptable for determining antifouling biocides in real water samples. Furthermore, the use of IL provides a simple, quick, less toxic, and ecologically favorable technique for determining the booster biocides in water samples.

## Figures and Tables

**Table 1:** Recoveries and RSDs of Irgarol 1051 and Sea-Nine 211 spiked in water samples (*n* = 3).

Sample		Deionized Water			Lake Water			Seawater		
Irgarol 1051	Spiked level (μg L^−1^)	0.02	0.1	1.0	0.02	0.1	1.0	0.1	1.0	5.0
	Recovery (%)	87.0	80.3	85.7	85.7	90.2	81.0	79.1	82.4	85.2
	RSD (%)	5.4	1.2	2.5	3.9	4.2	5.6	5.6	1.2	3.1
	LOQ, LOD (μg L^−1^)	0.02, 0.01			0.02, 0.01			0.1, 0.05		
Sea-Nine 211	Spiked level (μg L^−1^)	0.1	1.0	5.0	0.1	0.5	5.0	0.5	5.0	10.0
	Recovery (%)	86.9	90.3	84.7	84.4	83.3	80.6	78.7	86.1	89.3
	RSD (%)	7.5	4.5	2.7	5.0	4.1	3.1	1.7	6.7	4.6
	LOQ, LOD (μg L^−1^)	0.06, 0.02			0.06, 0.02			0.5, 0.1		

**Table 2:** Comparison of the IL-DLLME method with other procedures for the determination of antifouling biocides in water samples.

Method	Sample Amount (mL)	Extraction Solvent	Solvent Volume ^a^ (mL)	Extraction Time ^b^ (min)	Extraction Recovery (%)	LOD (μg L^−1^)	RSD%
SPE-GC-MS [53]	200	EA	15	46	42–95	0.0012–0.0015	<10
SPE-LC-MS/MS [54]	100	ACN	12	Not given	77–93	0.002	<8
SPME-GC-MS [55]	3	—	—	60	Not given	0.05–0.2	<20
SPE-LC-MS/MS [56]	1000	MeOH, DCM	9	200	80–120	0.001	<18
SPE-LC-MS/MS [57]	250	MeOH, DCM	8	25	78–120	0.0003–0.0027	<13
SPE-LC-QTOF/MS [58]	200	MeOH, DCM	8	60	79.7–119.2	Not given	17.7–27.7
LLE-GC-MS [59]	2000	DCM	50	Not given	70–120	0.001	30
SPE-GC-MS [60]	2000	EA, AC	15	145	>90	0.001	<10
SPE-LC-MS [61]	500	10 mM HAc MeOH	15	65	82.5–111	0.0002–0.001	3–5
SBSE-TD-GC-MS [20]	10	—	—	90	72–125	0.005–0.9	7–15
LLE-GC-MS [62]	1000	Toluene	1	60	73.55–120.28	0.00177–0.01242	1.64–4.87
MF-LPME-HPLC-UV [21]	300	Toluene	0.4	90	Not given	0.001–0.0048	<12
IL-DLLME method	5	[HMIm][PF6]	0.046	1	80–90	0.01–0.1	<8

^a^ Solvent consumption only in the extraction stage; solvent consumption in solvent exchanges not included. ^b^ Time employed in the extraction stage; any other operations were not included. GC-MS, gas chromatography-mass spectrometry; LC-MS/MS, liquid chromatography-tandem mass spectrometry; LC-QTOF/MS, liquid chromatography-quadrupole time-of-flight mass spectrometry; EA, ethyl acetate; ACN, acetonitrile; MeOH, methanol; DCM, dichloromethane; AC, acetone; and HAc, acetic acid.

**Table 3:** Basic information and chromatographic parameters of the analytes.

Analyte	Chemical Structure	Molecular Weight	Retention Time (min)	Mass Ions (*m*/*z*)	Fragmentor Voltage (V)
Irgarol 1051	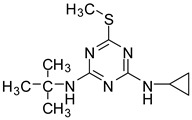	253.1	8.53	254.0 [M + H]^+^ 198.0 *	120 230
Sea-Nine 211	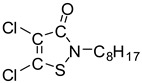	281.0	12.89	282.0 [M + H]^+^ 304.0 * [M + Na]^+^	90 100

* Quantitative ion.

## Data Availability

Data sharing is not applicable to this article.

## Sample Availability

Samples of the compounds are not available from the authors.
